# A comparative study to determine the association of gut microbiome with schizophrenia in Zhejiang, China

**DOI:** 10.1186/s12888-022-04328-w

**Published:** 2022-11-24

**Authors:** Fuyang Yan, Lehong Xia, Li Xu, Liyun Deng, Guolin Jin

**Affiliations:** The Second People’s Hospital of Lishui, Lishui, 323000 China

**Keywords:** Gut microbiome, 16S rRNA sequencing, Schizophrenia, Biomarker

## Abstract

**Background:**

With the rapid progress of high-throughput sequencing technology, characterization of schizophrenia (SZ) with underlying probing of the gut microbiome can explore pathogenic mechanisms, estimate disease risk, and allow customization of therapeutic and prophylactic modalities. In this study, we compared the differences in gut microbial diversity and composition between 50 SZ subjects and 50 healthy matched subjects in Zhejiang, China via targeted next-generation sequencing (16S rRNA amplicon).

**Results:**

Accordingly, the alpha diversity indices (observed species index, Shannon index, and Simpson index) of the gut microbiome in the healthy control group were higher than those in the SZ group. Additionally, principal coordinate analysis and non-metric multidimensional scaling of beta diversity revealed that patients with SZ clustered more tightly than healthy controls. At the phylum level, we found that the abundance of *Bacteroidetes* and *Proteobacteria* in the SZ group was significantly increased. At the genus level, the relative abundances of *Prevotella*, *Parabacteroides*, and *Sutterella* were significantly higher, whereas the abundances of *Faecalibacterium*, *Blautia*, *Lachnospira*, *Clostridium*, *Ruminococcus*, and *Coprococcus* were lower than those in the healthy control group. Further analyses revealed that *Succinivibrio*, *Megasphaera*, and *Nesterenkonia* may serve as potential biomarkers for distinguishing patients with SZ from those in the control cohort.

**Conclusions:**

This study profiled differences in gut microbiome diversity, taxonomic composition, and function between SZ and healthy cohorts, and the insights from this research could be used to develop targeted next-generation sequencing-based diagnoses for SZ.

**Supplementary Information:**

The online version contains supplementary material available at 10.1186/s12888-022-04328-w.

## Introduction

The human gut microbiota, a complex ecosystem comprising 100 trillion microorganisms (bacterial species, fungi, and viruses) carrying over three million genes (gut microbiome), can influence human physiology, behavior, and health [1, 2]. It changes over time depending on the host’s genetic predisposition, age, behavior, dietary habits, antibiotic use, and living environment. Microorganisms located in the human gastrointestinal tract carry various functionalities, including the absorption of nutrients and minerals, the constructive synthesis of short-chain fatty acids (SCFAs) and vitamins, and the production of microbial metabolites. Converging evidence suggests that differences in the human gut microbial community contribute to various diseases, such as liver diseases, diabetes, inflammatory bowel disease, multiple sclerosis, colorectal cancer, and neuropsychiatric disorders [3–8]. In addition, a smaller number of studies have revealed predicted or actual functional alterations in microbial genes or metabolic pathways directly or indirectly associated with these diseases. Therefore, understanding the communication or signaling pathways involved in the interactions between humans and microbiota is essential for investigating pathogenic mechanisms, estimating disease risk, allowing the customization of therapeutic modalities, monitoring therapeutic progress, and developing prophylactic strategies.

Since most intestinal microorganisms are difficult to culture, next-generation sequencing (NGS) platforms with high throughput, read length, sensitivity, specificity, precision, and accuracy have been widely applied to characterize microbial composition. To increase the sensitivity of microbial identification and overcome the dilemma of amplifying low levels of microbial sequences, the process of enrichment for target and interesting sequences (16S rRNA, 18S rRNA, 23S rRNA [9] and internal transcribed spacer) can be performed via polymerase chain reaction (PCR) before high-throughput sequencing [9–12]. Although targeted next-generation sequencing (tNGS) only directly characterizes bacterial taxonomy, it is a cost-effective option for exhaustively covering the biodiversity (measuring the maximal dynamic range of relative abundance) of many samples via minimal sequencing. Such a powerful alternative has strongly contributed to various novel discoveries in the past decade, helping us to survey and characterize the gut microbiome from the human gut, soil and oceans [13–15]. More importantly, bioinformatics analysis may be best suited to explore predicted or actual microbial functions and metabolic pathways.

Schizophrenia (SZ) is a serious psychiatric disorder with a global lifetime prevalence of 0.4% and a heritability of around 0.81 (confidence interval, 0.73–0.90) [16, 17]. The marked symptoms are mainly divided into five domains: hallucinations, delusions, disorganized thinking (speech), grossly disorganized, and abnormal human behaviors. Although the physiological phenomenon of SZ has not yet been explored or explained, patients diagnosed with SZ are frequently characterized by psychotic symptoms, poor social functioning, and a poor quality of life. For molecular diagnosis of SZ patients, accumulating evidence of NGS indicates that alterations in microbial diversity and taxonomic composition in the human gut are widely observed in SZ patients compared with healthy matched controls, which greatly promotes the development of biomarkers [18–20]. However, we are in the way of completely distinguishing normal microbiota from that present in SZ or recognizing how its substantial metabolic pathways act on health. In addition, there is intensive and extensive bidirectional signaling between the gut microbiota and central nervous system through the gut-brain axis [21]. This process strongly correlates with neuronal, endocrine, and immunological mechanisms, which allow the gut microbiota to influence various human psychiatric status and homeostasis. Thus, it is necessary to decipher the content, diversity, and function of the gut microbiota to evaluate therapeutic opportunities and strategies in patients with SZ.

In this study, we focused on investigating and characterizing the differences in gut microbial diversity and taxonomic composition between people with SZ and healthy people. In addition, we predicted the genetic potential of the gut microbiota and elucidated how functional differences affect the physiological processes within the human gut. We aimed to present a comprehensive insight into the predicted or actual pathogenic mechanism of the human gut microbiota of SZ, with a particular emphasis on the potential of microbe-based diagnostic biomarkers.

## Materials and methods

### Study population and sample collection

Briefly, 100 men between the ages of 20 and 58 years participated in this study. And 50 patients with SZ were recruited from Lishui City Second People’s Hospital (Zhejiang, China), and 50 healthy matched participants (NC group) were recruited from the same province. SZ was diagnosed according to symptoms including delusions, disorganization in the form of thought, hallucinations, impaired attention, loss of motivation, blunted emotional expression, and bizarre behaviour which based on the International Statistical Classification of Diseases and Related Health Problems, 10th Revision (ICD-10, World Health Organization). Symptoms must be present for at least one month, and the manifestation caused by other health conditions and substance or medication use was excluded. The patients were selected only if the severity of schizophrenia met the standard of ICD-10. Furthermore, the dosage of antipsychotic medication was stable within 3 months before sample collection. All eligible subjects without genetic disorders maintained a regular diet and aerobic exercise and refused to take antibiotics. Prior to the study, all eligible participants and their guardians received a full explanation of the study and provided written informed consent.

Both SZ and NC subjects were provided with Stool Collection Tube (Simgen, Hangzhou, China) and provided with the detailed manufacturer's protocol for collecting fresh fecal samples. All samples were stored at -80 °C.

### DNA extraction and sequencing

Before DNA extraction, the samples were pre-processed according to the 16S rRNA Earth Microbiome Protocol (earthmicrobiome.org). Then, gDNA was extracted from samples using the Omega Bio-tek Stool DNA Kit (Omega Bio-Tek, Doraville, CA, USA) according to the manufacturer’s instructions. Additional positive and negative DNA controls were included so that sample exclusion based on read counts could be calculated. Thereafter, the quality of the DNA was evaluated using agarose gel electrophoresis and PicoGreen assay (Thermo Fisher Scientific, Cleveland, OH, USA). Finally, the DNA was stored at − 20 °C before sequencing.

First, 341f (5’-CCTAYGGGRBGCASCAG-3’) and 806r (5’-GGACTACNNGGGTATCTAAT-3’) barcoded primers were designed using Oligo software (v7.0) [22], validated using Primer-BLAST (https://www.ncbi.nlm.nih.gov/tools/primer-blast/), and synthesized by Sangon Biotech (Shanghai, China). Second, 16 s amplicons were generated via PCR based on a method described in a previous work [23]. The quality of the amplicons was evaluated using agarose gel electrophoresis and PicoGreen assay before cleaning with the Agencourt AMPure XP PCR purification kit (Beckman Coulter, Brea, CA, USA). Amplicon library amplification was performed using Nextera XT DNA Sample Prep (Illumina, San Diego, CA, USA). Then, the amplicon library was cleaned once with AMPure beads, and DNA concentration was measured on a Qubit fluorometer (Invitrogen, Carlsbad, CA, USA). Paired-end sequencing was performed on a MiSeq platform (Illumina, San Diego, CA, USA) using the MiSeq Reagent Kit v3 (Illumina, San Diego, CA, USA), generating 300 bp reads per end. Raw sequencing files were uploaded and analyzed using the MiSeq Reporter (Illumina, San Diego, CA, USA) for further analysis.

### Bioinformatic data processing

The quality of raw sequencing data was assessed using FastQC v0.11.3 (http://www.bioinformatics.babraham.ac.uk/projects/fastqc). To generate high-quality sequencing data, low-quality reads and Illumina adapters were excluded using Trimmomatic v0.36 [24]. Paired reads were merged and assembled using FLASH v1.2.11 with default settings [25]. Operational taxonomic units (OTUs) were defined as sequences with at least 85% similarity against the Greengenes database using the UClust clustering algorithm (http://drive5.com/usearch/) following the close-reference method in QIIME v1.9.1 [26, 27]. Amplicon sequence variants (ASVs) were identified using the Divisive Amplicon Denoising Algorithm 2 [28]. Taxonomic assignments of ASV representative sequences were performed with a confidence threshold of 0.8–1.0, using a pre-trained Naive Bayes classifier, which was trained on the Ribosomal Database Project classifier v11.5 [29]. Ordination was performed using principal component analysis (PCA) with the vegan function in the R package v4.0.2.

To account for both abundance and evenness, alpha diversity analyses, including the Observed Species index, Shannon diversity index [30] and Simpson diversity index [31] were performed using QIIME v1.9.1 [27]. Beta diversity was calculated using Bray–Curtis dissimilarity [32] and unweighted UniFrac [32, 33]. To visualize the similarity between samples, output matrices were ordinated using principal coordinate analysis (PCoA) and visualized using EMPeror [34]. Non-metric multidimensional scaling (NMDS) was computed for each sample based on the total beta diversity using the R package v4.0.2 (vegan). Analysis of similarities (ANOSIM) and indicator value analysis were performed using R package v4.0.2 (vegan and labdsv), respectively. Venn diagrams illustrating the genera common to all samples were produced using the Venn program v1.6.16. For functional community profiling, Tax4Fun was evaluated using QIIME v1.9.1 with the SILVA database extension [27, 35]. The key was to compare the 16S rRNA gene sequencing data with the KEGG database to achieve functional annotation [36].

### Statistical analysis

Participant demographic and clinical characteristics were summarized and analyzed using the Student’s t-test and chi-square test for continuous and discrete variables, respectively. Group significance (Welch’s t-test) and *post-hoc* analyses (false discovery rate and Bonferroni correction) were performed to identify ASVs that differed in abundance between the healthy peri-implant sites and those with peri-implantitis. Statistics and plots were performed using R software v4.0.2. Statistical significance was set than 0.05.

## Results

### Sampling information

Based on the inclusion and exclusion criteria, 50 male patients with SZ and 50 healthy male individuals were recruited. The demographic and clinical characteristics of both the groups are presented in Table 1. There were no significant differences in age (*p* = 0.1311), weight (*p* = 0.1811), and body mass index (BMI, *p* = 0.4817). However, the NC group had a significantly greater height than the SZ group (*p* = 0.0460). In terms of BMI classification, there were statistical differences between the thin (BMI < 18.5, *p* = 0.0412) and obese (BMI ≥ 28, *p* = 0.0412) groups. Finally, the distribution of antipsychotic use of patients with SZ mainly consisted of risperidone (28%), clozapine (20%), olanzapine (40%), and quetiapine (24%), and 26% of them took two types of antipsychotic drugs during clinical therapy.

**Table 1:** Demographic and clinical characteristics of participants

	SZ groups (*n* = 50)	NC groups (*n* = 50)	t or χ2	*p* value
Age	39.9	37.2	1.522^a^	0.1311
Weight (kg)	67.2	69.6	1.347^a^	0.1811
Height (cm)	169.8	171.4	2.021^a^	0.046
BMI	23.3	23.7	0.7063^a^	0.4817
BMI classification [n (% BMI < 18.5)]	4 (8)	0 (0)	4.167^b^	0.0412
BMI classification [n (% 18.5 ≤ BMI < 24)]	27 (54)	27 (54)	0^b^	1
BMI classification [n (% 24 ≤ BMI < 28)]	15 (30)	23 (46)	2.716^b^	0.0993
BMI classification [n (% 28 ≤ BMI)]	4 (8)	0 (0)	4.167^b^	0.0412
Hepatitis B [n (%)]	1 (2)	0 (0)	1.010^b^	0.3149
Antibiotic use [n (% in past 3 months)]	0	0	-	-
Antipsychotic use	0	0	-	-
Risperidone [n (%)]	14 (28)	0 (0)	16.28^b^	5.00E-05
Clozapine [n (%)]	10 (20)	0 (0)	11.11^b^	8.60E-04
Olanzapine [n (%)]	20 (40)	0 (0)	25.00^b^	0
Quetiapine [n (%)]	12 (24)	0 (0)	13.64^b^	2.20E-04
Sulpiride [n (%)]	1 (2)	0 (0)	1.010^b^	0.3149
Aripiprazole [n (%)]	3 (6)	0 (0)	3.093^b^	0.0786
Perphenazine tablets [n (%)]	1 (2)	0 (0)	1.010^b^	0.3149

*BMI* Body mass index

^a^Means Student’s t-test

^b^Means chi-square test (χ2 value)

## Microbial diversity

A total of 8,911,266 reads (1,869,170,520 bases) were obtained from the healthy control group, while 50 microbiome samples of SZ subjects consisted of 5,816,276 reads (1,320,847,922 bases). After quality filtration, adapter reduction, and paired-read assembly, we obtained 4,376,659 raw tags, 4,321,992 effective tags, and 21,507 OTUs in healthy control group, ranging from 23,459 to 414,271, 23,392 to 409,960, and 115 to 844 for each sample. Additionally, there were 2,667,140 raw tags, 2,645,060 effective tags, and 12,230 OTUs in the SZ group, ranging from 19,503 to 68,895, 19,387 to 67,766, and 101 to 516 for each sample, respectively. Both the rarefaction curve (Figure S1 A) and rank abundance curve (Figure S1 B) confirmed the validity of the high-throughput sequencing data and revealed that the abundance of the microbial community varied depending on the sample of individuals.

**Fig. 1:**
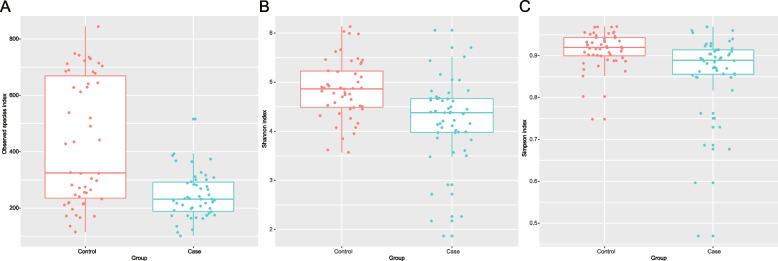
Alpha-diversity indices (**A**: Observed Species index, **B**: Shannon index, **C**: Simpson index) for the OTUs in the gut microbiota of SZ and NC groups

**Fig. 2:**
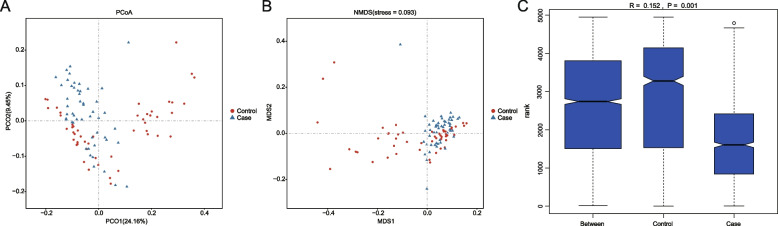
Unweighted UniFrac PCoA (**A**) and two-dimensional NMDS (**B**) plots comparing sample distribution between the two cohorts. **C** Unweighted UniFrac ANOSIM test for the16S rRNA gene. Samples were compared using peak height as a measure of abundance. Control indicates the NC group. Case indicates the SZ group

To characterize the richness and diversity of the microbial community, we calculated alpha indices for each sample. There were significant changes in the observed species index between the SZ and NC groups (Fig. 1A, *p* = 6.88e-07), Shannon index (Fig. 1B, *p* = 5.94e-05), and Simpson index (Fig. 1C, *p* = 6.85e-04) of alpha diversity. Conversely, OTU-based beta diversity is a comparative analysis of microbial community composition between samples. We observed that the PCoA (Fig. 2A) and NMDS-based map (Fig. 2B, stress = 0.093) of unweighted UniFrac metrics revealed that SZ subjects were tightly clustered when NC subjects formed distinct clusters (within-group distance comparison). In addition, some NC clusters were close to those of the SZ group. Moreover, the ANOSIM analysis (Fig. 2C) indicated that the microbial community structure was significantly different (unweighted UniFrac, *R* = 0.152, *p* = 0.001) between the two groups.

### Differences in taxonomic composition

**Fig. 3:**
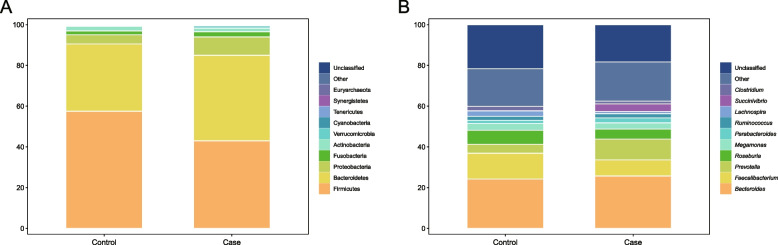
Microbial composition at phylum level (**A**) and genus level (**B**). Control indicates the NC group. Case indicates the SZ group

To better understand OTU information and taxonomic annotation, tags and OTUs were calculated and summarized. As is shown in Fig. 3A, the predominant bacteria in the NC group were *Firmicutes* (57.43%), *Bacteroidetes* (33.08%), *Proteobacteria* (4.55%), *Actinobacteria* (2.31%), and *Fusobacteria* (1.83%), whereas the SZ cohort was dominated by *Firmicutes* (42.93%), *Bacteroidetes* (42.03%), *Proteobacteria* (9.04%), *Fusobacteria* (2.55%), and *Actinobacteria* (1.72%). When the relative abundances of bacterial phyla were compared (Figure S2A), *Bacteroidetes* (*p* = 4.11e-03) and *Proteobacteria* (*p* = 0.0371) were found to be more abundant in the SZ than in the NC group. In terms of *Firmicutes* levels, the SZ group showed a significant decrease (*p* = 3.98e-05) compared to the NC group. At the genus level (Fig. 3B), the NC group was mainly assigned to *Bacteroides* (24.26%), *Faecalibacterium* (12.59%), *Roseburia* (6.89%), *Prevotella* (4.43%), *Megamonas* (3.31%), *Blautia* (3.13%), *Lachnospira* (2.58%), *Clostridium* (2.30%), *Ruminococcus* (1.99%), and *Coprococcus* (1.81%). The most abundant genera in the SZ group were *Bacteroides* (25.66%), followed by *Prevotella* (10.24%), *Faecalibacterium* (7.95%), *Roseburia* (4.93%), *Succinivibrio* (3.68%), *Megamonas* (2.96%), *Parabacteroides* (2.49%), *Dialister* (2.00%), *Sutterella* (1.67%), and *Clostridium* (1.33%). Compared to healthy cohort (Figure S2B), the relative abundance of *Prevotella* (*p* = 0.0157), *Parabacteroides* (*p* = 0.0342), and *Sutterella* (*p* = 0.0365) was significantly higher in the SZ cohort. However, *Faecalibacterium* (*p* = 4.25e-03), *Blautia* (*p* = 3.04e-05), *Lachnospira* (*p* = 6.36e-03), *Clostridium* (*p* = 0.0287), *Ruminococcus* (*p* = 0.0380), and *Coprococcus* (*p* = 0.0258) levels were higher in the healthy cohort.

**Fig. 4:**
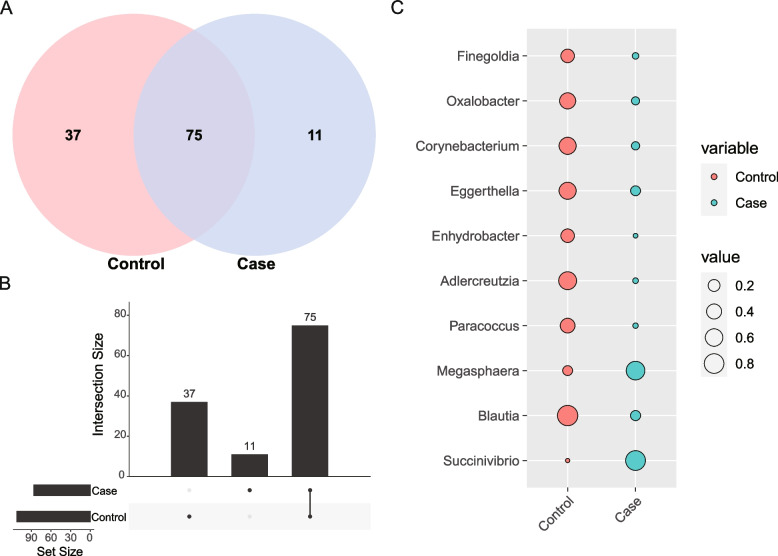
**A** Venn diagram of shared and specific genera. **B** Venn diagram of upset. **C** Indicator value of specific genera. Control indicates the NC group. Case indicates the SZ group

The Venn diagram illustrates the distribution of shared and specific genera to identify the candidate microbial biomarkers. As is shown in Fig. 4A and 4B, all individuals, irrespective of SZ patients and healthy persons, had in common 73 genera of the total members that were consistently detected. In addition, we calculated the indicator value to identify candidate biomarkers for microbiological diagnosis. *Succinivibrio* (*p* = 0.001), *Megasphaera* (*p* = 0.001), and *Nesterenkonia* (*p* = 0.005) were more enriched in the SZ group, whereas *Blautia* (*p* = 0.001), *Paracoccus* (*p* = 0.001), *Adlercreutzia* (*p* = 0.001), *Enhydrobacter* (*p* = 0.001), *Eggerthella* (*p* = 0.002), *Corynebacterium* (*p* = 0.002), *Oxalobacter* (*p* = 0.002), and *Finegoldia* (*p* = 0.005) in healthy control subjects (Fig. 4C).

### Functional differences of microbiome

**Fig. 5:**
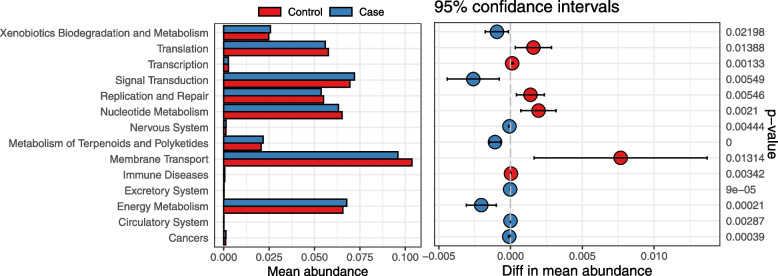
Significant differences (*P* < 0.05) in KEGG pathways for gut microbiota in SZ (case) and NC (control) groups. The bars represent the average relative abundance of functional pathways, having significant differences between the two groups, with 95% confidence interval distribution and *p* value shown on their right

**Fig. 6:**
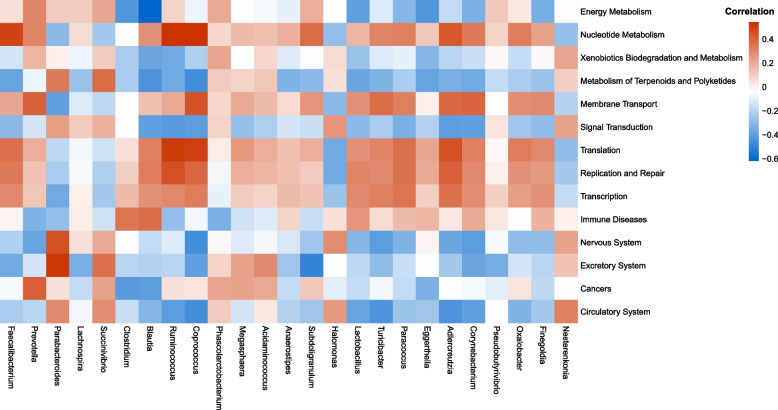
Heat-map of correlation between the relative abundances of the alter genera and the differentially relative abundance metabolism pathway. Colors indicate the Pearson correlation coefficients

Tax4Fun analysis was performed to reveal and explore the differences in the function of the gut microbiome between the SZ and NC groups. As is shown in Fig. 5, the metabolism of terpenoids/polyketides (*p* = 4.37e-07), excretory system (*p* = 9.50e-05), energy metabolism (*p* = 2.08e-04), cancers (*p* = 3.93e-04), circulatory system (*p* = 2.87e-03), nervous system (*p* = 4.44e-03), signal transduction (*p* = 5.49e-03), and xenobiotic biodegradation/metabolism (*p* = 0.0220) in the SZ group showed an upward trend compared to that in the NC group. However, there were significant decreases in transcription (*p* = 1.33e-03), nucleotide metabolism (*p* = 2.10e-03), immune diseases (*p* = 3.42e-03), replication/repair (*p* = 5.46e-03), membrane transport (*p* = 0.0131), and translation (*p* = 0.0139) in the SZ group. Next, we analyzed the correlation between the relative abundance of the altered genera and differentially functional pathways. *Faecalibacterium*, *Ruminococcus*, *Coprococcus*, *Adlercreutzia*, *Blautia*, and *Paracoccus* were positively associated with nucleotide metabolism, transcription, replication/repair, and translation (Fig. 6). Meanwhile, *Parabacteroides* was positively correlated with the metabolism of terpenoids/polyketides, the nervous system, excretory system, and circulatory system when *Prevotella* had a positive effect on cancers. In contrast, *Coprococcus*, *Corynebacterium*, and *Adlercreutzia* were negatively associated with signal transduction, the nervous system, and the circulatory system. Moreover, *Blautia* was negatively correlated with energy metabolism and cancer.

## Discussion

In our study, microbial diversity and composition in the gut of patients with SZ and healthy control subjects from Zhejiang, China, were evaluated using tNGS of the V3 and V4 regions of the 16S rRNA gene with bioinformatics analysis. Our first main finding was that the microbial diversity of the human gut was altered between the SZ and NC groups. The alpha diversity indices (observed species index, Shannon index, and Simpson index) of the gut microbiome in the NC group were higher than those in the SZ group, indicating a higher richness and diversity of the microbial community in healthy subjects. It is typically observed that the scores of alpha-diversity in psychiatric populations decrease in SZ patients [37, 38]. Generally, a high index of alpha diversity is considered as a marker of a healthy status. In summary, the lower alpha diversity suggested an overall abnormal microbial ecology within patients here and was linked to a range of chronic human diseases [39]. Additionally, beta diversity metrics were considered using nonphylogenetic methods. The resultant beta diversity showed that patients with SZ clustered tightly when the clusters of NC subjects were spread more widely across the PCoA and NMDS spaces. Therefore, the OTU definition and taxonomic annotation were performed for further analyses.

The intestinal microbiota community structure at the phylum and genus levels was mainly analyzed. Regardless of age, weight, height, BMI, and BMI classification, the gut microbial composition of normal people and patients with SZ mainly consisted of five major phyla: *Firmicutes*, *Bacteroidetes*, *Proteobacteria*, *Actinobacteria*, and *Fusobacteria*. This result is consistent with previous researches [18, 40, 41]. However, the most important alteration was that the relative abundance of *Bacteroidetes* (genus: *Prevotella* and *Parabacteroides*) and *Proteobacteria* (genus: *Sutterella*) was obviously higher in patients with SZ than in healthy controls. In contrast, we found that the proportion of *Firmicutes* (genus: *Faecalibacterium*, *Blautia*, *Lachnospira*, *Ruminococcus*, and *Coprococcus*) *and Actinobacteria* (genus: *Corynebacterium* and *Adlercreutzia*) was lower in SZ subjects. An increasing number of studies have validated that *Faecalibacterium*, *Blautia*, *Lachnospira*, *Ruminococcus*, *Coprococcus*, *Corynebacterium*, and *Adlercreutzia* are beneficial phylotypes. The relationship between bacterial abundance and function was analyzed and discussed. Our results revealed that *Faecalibacterium*, *Blautia*, *Ruminococcus*, *Coprococcus*, *Adlercreutzia*, and *Paracoccus* regulated diverse molecular processes, such as nucleotide metabolism, transcription, replication/repair, and translation. As is widely known, nucleotide metabolism is required for nucleic acid synthesis, DNA proliferation, DNA repair, and RNA production to maintain genome stability. Besides, RNA is translated into proteins with the correct structure and is involved in cell proliferation, maintenance, repair, and regulation at different stages of the cell cycle.

The decrease in *Coprococcus*, *Corynebacterium*, and *Adlercreutzia* resulted in an upward trend in signal transduction, nervous system, and circulatory system in SZ subjects. More seriously, the decreased level of *Blautia* caused an upregulated status of cancer. It has been reported that the decrease in *Blautia*, *Lachnospira Coprococcus*, *Corynebacterium*, and Adlercreutzia may be caused by antipsychotic medication and are strongly associated with a reduction in SCFAs [42]. SCFAs are capable to modulate a variety of immune and epigenetic pathways, such as barrier function in intestinal epithelial cells, obesity-associated inflammation, release of interleukin-6 and tumor necrosis factor-α from macrophages, cytokine-associated nuclear factor kappa-B signaling pathway [43] and inhibition of histone deacetylase [43–46]. These biological pathways have also been examined and validated to be dysregulated in SZ. Although SCFAs have anti-inflammatory properties, the central role of SCFAs in the brain and their relationship with neurobiological factors and pathways, including neurotransmitter circuits, neurotrophic factors, and other brain metabolites, remains largely unknown.

The most important limitation of this study was its small sample size, which must be acknowledged. For example, only 100 male subjects from the same area participated in this study, and one microbiome sample was tested per individual. Generally, microbial diversity and composition fluctuate across sex, area, time, and other factors [23, 47]. Likewise, we did not match the gut microbiome or control for smoking prevalence in our analyses. Patients with SZ are markedly prone to smoke tobacco and it has been suggested that biological factors may underlie the association between this disorder and tobacco use. Moreover, mechanistic studies on animal and cellular aspects are absent. All these statuses limited the extent to which we could statistically explore predicted or actual confounds on the differences in the microbiome and the power of correlational analyses between microbiota and functional differences. Therefore, these results should be considered as preliminary.

### Limitations

Although there are several limitations to our study, we still make a critical contribution to the monitoring of SZ. Based on the taxonomic community, the Venn diagram showed that 11 genera had the potential to serve as vital diagnostic biomarkers for distinguishing SZ. To further validate the biomarkers that contributed significantly to the prediction performance, we calculated the indicator value and performed Welch’s t-test. The predictive model for *Succinivibrio*, *Megasphaera*, and *Nesterenkonia* at the genus level revealed remarkable discriminating power. This microbial diagnostic strategy is highly accurate and efficacious. In the future, we might be able to assist psychiatric physicians in diagnosing SZ and predicting disease progression by measuring the tNGS-based microbiota.

### Future suggestions

In conclusion, we have confirmed that there are some differences in the composition and function of the gut microbiome between patients with SZ and healthy individuals, and the insights from this research could be used to develop a tNGS-based diagnosis for SZ. In the future, it is likely that microbial biomarkers will become fast and highly sensitive tools for the detection and diagnosis of SZ disease. Further well-designed trials are needed to validate the results and conclude causal associations using animal models in a clinical setting.

## Supplementary Information

**Additional file 1: Figure S1.** (A) Rarefaction curve analysis of archaeal 16S rRNA gene clone libraries. (B) Rank abundance curves of archaeal 16S rRNA gene clone libraries. Sample color codes are presented in the legend. **Figure S2.** Microbial composition and abundance at phylum level (A) and genus level (B) for gut microbiota in SZ (Case) and NC (Control) groups. The bars represent the average relative abundance of each genera, having significant differences between the two groups, with 95% confidence interval distribution and *p* value shown on their right.

## Data Availability

The datasets used and/or analysed during the current study were submitted to the NCBI Bioproject repository, [Accession number PRJNA880407].

## Abbreviations

BMI: Body mass index
NGS: Next-generation sequencing
PCR: Polymerase chain reaction
SZ: Schizophrenia
SCFAs: Short-chain fatty acids
tNGS: Targeted next-generation sequencing
