# Supplementary material for: A comparative study to determine the association of gut microbiome with schizophrenia in Zhejiang, China
Source: BMC Psychiatry. 2022 Nov 24;22:731. doi: 10.1186/s12888-022-04328-w (PMC9694861; doi:10.1186/s12888-022-04328-w)
Supplement: Supplementary file 1 — Additional file 1: Figure S1. (A) Rarefaction curve analysis of archaeal 16S rRNA gene clone libraries. (B) Rank abundance curves of archaeal 16S rRNA gene clone libraries. Sample color codes are presented in the legend. Figure S2. Microbial composition and abundance at phylum level (A) and genus level (B) for gut microbiota in SZ (Case) and NC (Control) groups. The bars represent the average relative abundance of each genera, having significant differences between the two groups, with 95% confidence interval distribution and p value shown on their right. [file 12888_2022_4328_MOESM1_ESM.ppt]

## Slide 1
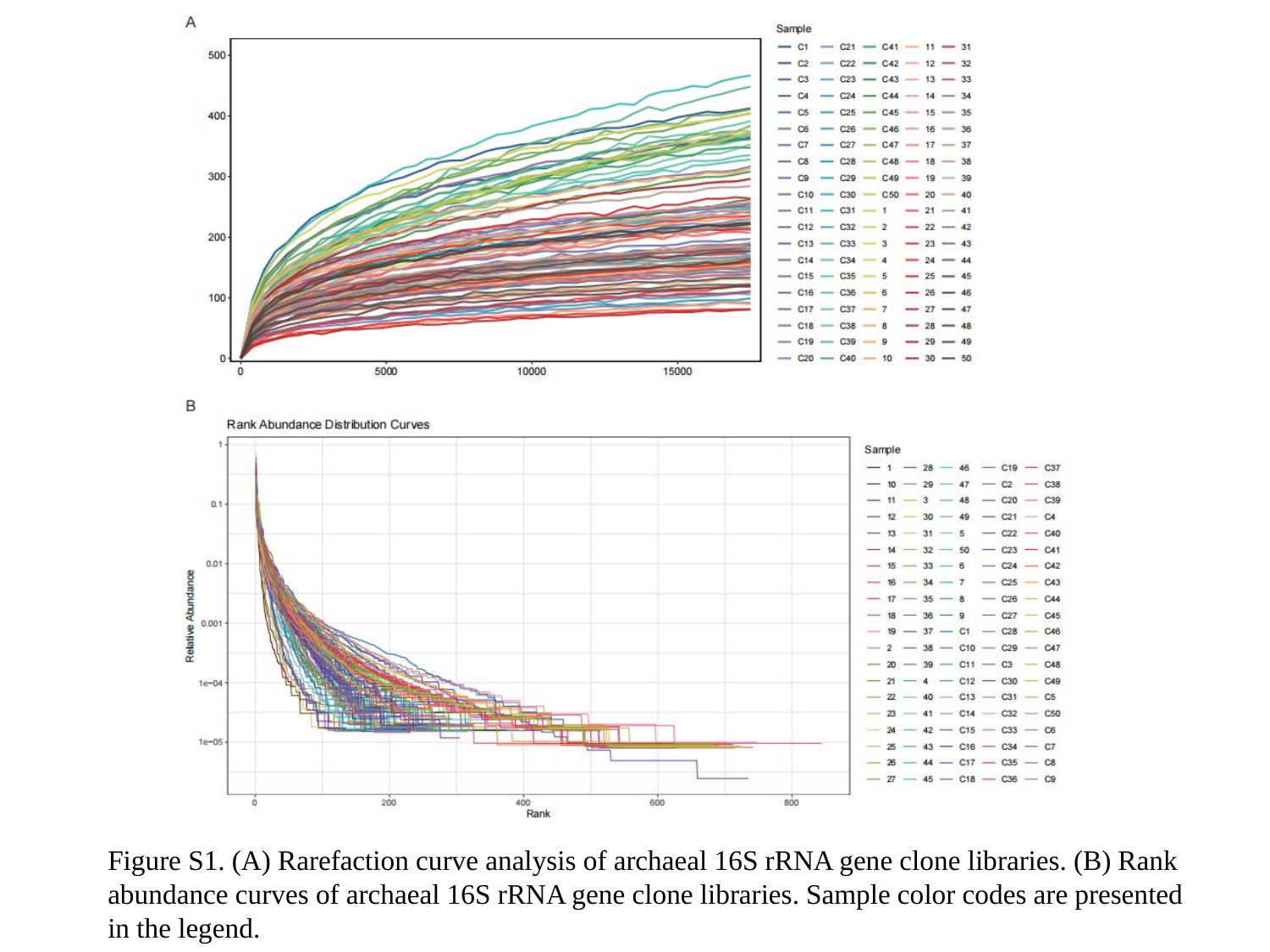

Figure S1. (A) Rarefaction curve analysis of archaeal 16S rRNA gene clone libraries. (B) Rank abundance curves of archaeal 16S rRNA gene clone libraries. Sample color codes are presented in the legend.

## Slide 2
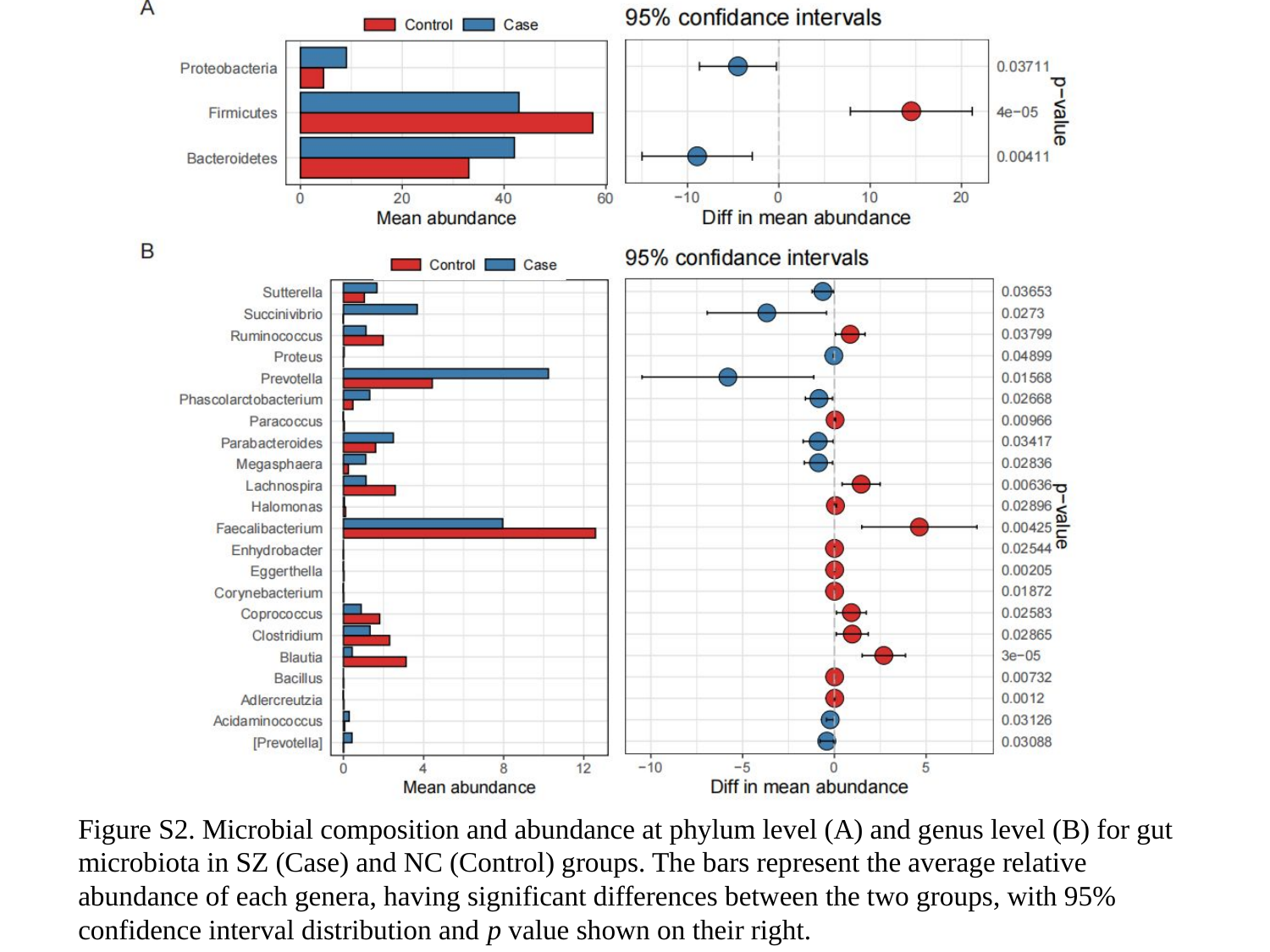

Figure S2. Microbial composition and abundance at phylum level (A) and genus level (B) for gut microbiota in SZ (Case) and NC (Control) groups. The bars represent the average relative abundance of each genera, having significant differences between the two groups, with 95% confidence interval distribution and p value shown on their right.
